# Risk factors for colonization and infection with multidrug-resistant *Pseudomonas aeruginosa* in intensive care unit: protocol for a systematic review and meta-analysis

**DOI:** 10.1186/s13643-022-02143-8

**Published:** 2022-12-13

**Authors:** Serge Eyebe, Hugues C. Nana-Djeunga, Magellan Guewo-Fokeng, Guy Sadeu Wafeu, Marius Zambou Vouking, Salomon Massoda, Christine D. Evina, Aurelia Condomat Zoumabo, Pierre Ongolo-Zogo, Jean-Ralph Zahar

**Affiliations:** 1 UMR 1137, Infection, Antimicrobiens, Modélisation, Evolution, Université Sorbonne Paris Nord, Paris, France; 2 Department of Public Health, Faculty of Medicine and Biomedical Sciences, University of Yaoundé I, Yaoundé, Cameroon; 3 Centre for Research on Filariasis and other Tropical Diseases (CRFilMT), Yaoundé, Cameroon; 4 Department of Biochemistry, Faculty of Medicine and Biomedical Sciences, University of Yaoundé I, Yaoundé, Cameroon; 5 Centre for Development of Best Practices in Health (CDBPH), Yaoundé, Cameroon; 6 Ministry of Public Health, Yaoundé, Cameroon

**Keywords:** Antibiotic resistance, Resistant *Pseudomonas aeruginosa*, multidrug resistance, Infection, Intensive care unit, Risk factors

## Abstract

**Background:**

Infection with resistant *Pseudomonas aeruginosa (RPA)* in the intensive care unit (ICU) is known to be either endogenous or exogenous or both, but the roles of each of these contamination routes are yet to be clarified. Data regarding prevalence, risk factors, and environmental factors associated with RPA in ICU are very scanty and even when they exist, they seem to be contradictory. So, there is a strong interest in understanding both individual and environmental factors associated with RPA infection. This systematic review aims to investigate individual and environmental factors associated with the colonization and infection with RPA in ICU.

**Methodology:**

MEDLINE (Pubmed), EMBASE (OVID), the Cochrane Library (Wiley), Web of Science, CINAHL (EBSCOHost), and LILACS (BIREME) will be searched from inception onwards. Grey literature will be identified through Google Scholar and Open Grey. Two reviewers will independently screen all citations, abstracts, and full-text articles. Potential conflicts will be resolved through discussion. Methodological quality including bias will be appraised using appropriate approaches. A narrative synthesis will describe the quality and content of the epidemiological evidence. Prevalence, odds ratio, relative risk, and hazard radio with their respective 95% confidence intervals will be calculated. A meta-analysis of data extracted from eligible studies with similar populations and RPA testing will be performed. The analysis will evaluate factors influencing the estimates. A random effect model will be used to summarize effect sizes.

**Discussion:**

Two contrasting hypotheses on risk factors of acquisition, colonization, and infection of RPA are being debated, especially in a context where available data are scanty or exhibit high discrepancy. Indeed, most of the reviews have been focalized on hospitalized patients, and not in ICU, and few of them address the issue of environmental factors. To fill that gap, this review will combine both analyses of individual and environmental risk factors using prevalence studies in ICU and evaluation of different methodologies. These two hypotheses will be tested and challenged and could serve as a basis for a more in-depth study to fill the methodological gaps that will be identified as part of this current review.

**Systematic review registration:**

This protocol has been submitted to the Prospective Register of Systematic Reviews (PROSPERO) and the registration number attributed was CRD42021233832 of 07 March 2021.

## Background

*Pseudomonas aeruginosa* (PA) is a ubiquitous gram-negative bacterium with minimal survival requirements in the environment.

It has a remarkable ability to colonize environmental surfaces even when they are most hostile. PA can cause infections in patients, particularly when they are immunocompromised [1]. Infections by PA are among the major pathogens involved in healthcare-associated infections in intensive care units (ICUs) [2]. The prevalence of hospital-acquired PA carriage was 15.3% in the adult French population hospitalized in ICUs [3]. Furthermore, the resistance of *P. aeruginosa* to various antibiotics is increasing as is the case with the global burden of antibiotics resistance [4]. A cohort study conducted in Spain reported more than 80% of isolates susceptible only to amikacin and colistin and 9% only to colistin [5]. Infections due to resistant *Pseudomonas aeruginosa* (RPA) has been associated with increased morbidity and mortality rates [6, 7], increased length of stay in ICU, a high number of surgeries, and invasive procedures [6–8]. Although existing literature demonstrates increased costs related to resistant Gram-negative infections, data focused on *P. aeruginosa* are limited and somewhat inconsistent [8]. Infections with PA in ICU are known to be either endogenous or exogenous or both, but the role of each of these contamination routes is yet to be clarified [9]. Endogenous transmission has been widely described [2, 10], the main source of contamination being the intestinal microbiota under antibiotic pressure [10]. Exogenous transmission occurs from hydrous environments (tap, sink...), invasive medical devices, and the patients and health personnel through their hands [11, 12]. Most studies lean towards a predominance of the endogenous source, the exogenous source being more often incriminated in outbreaks [3, 11, 13]. Moreover, the source of RPA in ICU is not well established, as well as factors associated with the carriage, colonization, and, infection [9]. Data regarding prevalence, risk factors, and environmental factors associated with RPA in ICU are very scanty and even when they do exist, they seem contradictory. For example, a systematic review performed in 2018 demonstrated that there are considerable gaps and inconsistencies in knowledge regarding risk factors associated with RPA or identifying subgroups of patients at increased risk of acquisition of RPA in hospitals [12]. Unfortunately, this systematic review did not specifically address the issue in intensive care units and was limited to endogenous risk factors [12]. There is, therefore, growing interest in understanding the individual and environmental factors associated with resistant *P. aeruginosa*, particularly in intensive care units where high rates of morbidity and mortality are being observed [6, 9].

The main objective of this systematic review will be to investigate individual (endogenous) and environmental (exogenous) factors associated with the colonization and infection with resistant *Pseudomonas aeruginosa* in intensive care units. The secondary objectives of this study will be to (i) estimate the prevalence of multidrug-resistant *P. aeruginosa* colonization in ICU, (ii) describe the main risk factors of acquisition and colonization with multidrug-resistant *P. aeruginosa* in ICU, and (iii) describe the main environmental sources of acquisition and colonization with multidrug-resistant *P. aeruginosa* in ICU.

## Methods/design

This systematic review and meta-analysis protocol are reported according to the Preferred Reporting Items for Systematic Review and Meta-Analysis Protocols (PRISMA-P) checklist provided as an additional file [14].

### Eligibility criteria

#### Type of studies to be included

This review will be included cross-sectional studies, case-control studies (nested or not), and cohort studies (either prospective, retrospective, or ambi-directional) conducted in intensive care units (ICUs). For the meta-analysis, only articles in which risk factors of MDR *Pseudomonas aeruginosa* acquisition/infection could be assessed will be selected. Studies without data on MDR-PA will be excluded. For multi-disease studies, when MDR-PA data will be not available separately in isolation, these studies will be excluded.

#### Type of participants

Studies include all patients, admitted to an ICU, whatever the reason for admission and the origin (community, another hospital, or another unit of the same hospital) of patients. No specific definition of ICU will be applied, and all studies which authors reported as having been conducted in an ICU will be considered. We will also include studies where it will possible to identify patients with specimens collected and tested for *P. aeruginosa* (positivity and antimicrobial susceptibility) during hospitalization and/or samples collected from the ICU patient’s environment (including staff hands and gloves) for *P. aeruginosa* testing. No specific restrictions on the type of patients will be applied.

### Outcomes

#### The primary outcome

Factors of acquisition of MDR-PA. Since the year 2000, the most common definition of MDR used for gram-positive and gram-negative bacteria is ‘resistant to three or more antimicrobial classes [15].

#### Secondary outcomes

(i) Risk factors of carriage. The carriage was defined as positive screened specimens. These specimens could be collected from rectal swabs, nasal swabs or tracheal aspiration, or others [16].
(ii) Risk factors of colonization. Colonization was defined as positive clinical specimens and infection as positive clinical specimens with clinical signs confirming infection.

### Search strategy

Six databases will be searched from inception onward, namely MEDLINE (Pubmed), EMBASE (OVID), the Cochrane Library (Wiley), Web of Science, CINAHL (EBSCOHost), and LILACS (BIREME). We will also search in grey literature through.

A search strategy using medical subject headings (MeSH) and text words related to *Pseudomonas aeruginosa*, intensive care units, carriage, colonization, or infection will be used (Table 1). For studies that will not be found via an internet search, the authors will be directly contacted to obtain them. Also, when a full text will be not available online, the author will be asked by email or telephone to provide it.

**Table 1:** Search strategy

Search	Query
#1	*Pseudomonas aerugnosa* [tw] or Pseudomonas [tw] or *P. aeruginosa* [tw] or Pseudomonadaceae [tw] or Gram-negative bacteria [tw] or gram negative [tw] or bacteria [tw] or Pseudomonas aerugnosa [mesh] or Pseudomonas [mesh]
#2	Intensive care unit [tw] or intensive care units [tw] or ICU [tw] or ICUs [tw] or intensive care [tw] or intensive care units [mesh] or respiratory care units [mesh]
#3	Carriage [tw] or colonization [tw] or colonisation [tw] or acquisition factors [tw] or infection [tw] or contamination [tw] or infected patients [tw] or asymptomatic Infections [mesh] or infections [mesh]
#4	Water [tw] or water tap [tw] or tap [tw] or bed [tw] or gloves [tw] or hands [tw] or environmental [tw] or environment [tw] or exogenous factors [tw] or Sink [tw] or care material [tw] or drains [tw] or surfaces water [mesh] or beds [mesh] or protective gloves [mesh] or environment [mesh]
#5	#3 or #4
#6	#1 and #2 and #5
#7	Date: from inception to December 31st, 2020
#8	No language restriction
#9	#6 and #7 and #8

### Study selection

The search will be conducted by an experienced information specialist, with no language restrictions. For studies published in a language other than English, Deepl translate will be used for translation. The result of electronic searches will be uploaded to Rayyan software for duplicate identification and removal. After this phase, two reviewers will independently screen titles and abstracts of all studies yielded by the search strategy. Firstly, they will screen the studies according to the inclusion and exclusion eligibility criteria. Then, the full text of these selected studies will be retrieved for a second analysis to decide whether the study should be finally included or not. Disagreements between the two reviewers will be resolved through discussion and consensus. PRISMA-P flow diagram template will be used to describe the number of articles retrieved and screened at each step [14].

### Data extraction

An online google form will be used to extract data from each included study by two independent reviewers. The following data will be extracted from each report:

- Study characteristics: first author name, year of publication, number of recruitment centers, study design, and sampling method.
- Participants: number of participants, region (s) and country/countries from which participants were recruited, study eligibility criteria, reasons for admission in ICU, age, gender, and duration of hospitalization.
- *P. aeruginosa* carriage, colonization, or infection data: number of persons who acquired MDR-PA, number of persons who already had MDR-PA, antibiotic tested, number and type of environmental specimens.
- Factors associated with resistant *P. aeruginosa*: all factors reported in studies will be collected in “authors’ own words” and with their corresponding effect estimates (odds ratios, relative risk, or hazard ratios).

### Data synthesis and management

Firstly, a descriptive analysis will be performed on the characteristics of studies, persons included, and risk factors obtained. Secondly, prevalence, odds ratio, relative risk, and hazard ratio with their 95% confidence intervals will be calculated from the compiled data. Thirdly, a meta-analysis of case-control and cohort studies with a similar population, same parameters, and *P. aeruginosa* testing will be performed. The analysis will evaluate factors influencing the estimates. An analysis by a sub-group of endogenous factors and by subtypes of exogenous factors will be performed. A random effect model will be used to summarize effect sizes. Heterogeneity between combined studies will be tested using the standard Chi-square test with the Q statistic (*p* <0.10 statistically significant). The extent of heterogeneity will be quantified using the I2 statistics; I2>50% will be deemed as representing substantial inconsistency or significant statistical heterogeneity. Where statistical pooling will be not possible, results will be presented in a synthetic narrative form. All the analyses will be carried out using Stata version 15 (StataCorp, College Station, TX).

### Quality of studies and evidence assessment

The methodological quality of studies will be assessed using the National Heart, Lung, and Blood Institute critical appraisal tools for each corresponding study design (cross-sectional, case-control, cohort). Potential bias in the design, conduct, and analysis of each study will be appraised by two independent reviewers. The confidence in evidence will be discussed among the authors.

## Discussion

This current review will have the specificity that it will include only studies conducted in intensive care units. Another specificity of this present review is that it will focus on *Pseudomonas aerugnisosa* by including not only endogenous but also exogenous acquisition factors. Indeed, most of the reviews have been focalized on hospitalized patients, and not in ICU, and few of them address the issue of environmental factors. This ongoing review will combine both analyses of individual and environmental risk factors using prevalence study in ICU, assessing of risk factors, and evaluation of different methodologies. These two hypotheses will be tested and challenged and could serve as a basis for a more in-depth study to fill the methodological gaps that will be identified as part of this current review. This review will assess a great number of articles because the search will be based on open keywords. We will compare findings between this ongoing review and past reviews [8, 12].

This review will allow us to identify risk factors endogenous or exogenous of colonization or acquisition by MDR PA. This could be useful in identifying patients at high risk for MDR *P. aeruginosa* that may benefit from alternate empiric treatment.

The high level of heterogeneity expected between the articles to be evaluated raises concerns about the production of evidence-based and the applicability of the review results.

## Data Availability

The datasets used and/or analyzed during the current study will be available and could be shared with the readers.

## Abbreviations

CDBPH: Centre for Development of Best Practices in Health
CRFilMT: Centre for Research on Filariasis and other Tropical Diseases
GRADE: Grading of Recommendations Assessment, Development, and Evaluation
ICU(s): Intensive care unit(s)
PRISMA-P: Preferred Reporting Items for Systematic Review and Meta-Analysis Protocols
PROSPERO: Prospective Register of Systematic Reviews
